# Noisecut: a python package for noise-tolerant classification of binary data using prior knowledge integration and max-cut solutions

**DOI:** 10.1186/s12859-024-05769-8

**Published:** 2024-04-20

**Authors:** Moein E. Samadi, Hedieh Mirzaieazar, Alexander Mitsos, Andreas Schuppert

**Affiliations:** 1 Institute for Computational Biomedicine, RWTH Aachen University, Aachen, Germany; 2 Process Systems Engineering (AVT.SVT), RWTH Aachen University, Aachen, Germany

**Keywords:** Hybrid mechanistic/data-driven modeling, Binary data, Overfitting, Max-cut problem, Noise-tolerant classification

## Abstract

**Background:**

Classification of binary data arises naturally in many clinical applications, such as patient risk stratification through ICD codes. One of the key practical challenges in data classification using machine learning is to avoid overfitting. Overfitting in supervised learning primarily occurs when a model learns random variations from noisy labels in training data rather than the underlying patterns. While traditional methods such as regularization and early stopping have demonstrated effectiveness in interpolation tasks, addressing overfitting in the classification of binary data, in which predictions always amount to extrapolation, demands extrapolation-enhanced strategies. One such approach is hybrid mechanistic/data-driven modeling, which integrates prior knowledge on input features into the learning process, enhancing the model’s ability to extrapolate.

**Results:**

We present NoiseCut, a Python package for noise-tolerant classification of binary data by employing a hybrid modeling approach that leverages solutions of defined max-cut problems. In a comparative analysis conducted on synthetically generated binary datasets, NoiseCut exhibits better overfitting prevention compared to the early stopping technique employed by different supervised machine learning algorithms. The noise tolerance of NoiseCut stems from a dropout strategy that leverages prior knowledge of input features and is further enhanced by the integration of max-cut problems into the learning process.

**Conclusions:**

NoiseCut is a Python package for the implementation of hybrid modeling for the classification of binary data. It facilitates the integration of mechanistic knowledge on the input features into learning from data in a structured manner and proves to be a valuable classification tool when the available training data is noisy and/or limited in size. This advantage is especially prominent in medical and biomedical applications where data scarcity and noise are common challenges. The codebase, illustrations, and documentation for NoiseCut are accessible for download at https://pypi.org/project/noisecut/. The implementation detailed in this paper corresponds to the version 0.2.1 release of the software.

**Supplementary Information:**

The online version contains supplementary material available at 10.1186/s12859-024-05769-8.

## Background

Binary-represented data arise in many clinical applications [1, 2]. Binary endpoints, which have two possible outcomes such as success/failure or present/absent, are commonly used in clinical trials to evaluate the effectiveness and safety of treatments [3]. Binary data also emerge in the context of the International Classification of Diseases (ICD) codes, which represent the presence of distinct medical diagnoses, conditions, and procedures [4]. Moreover, binary outcomes often result from longitudinal data analysis in clinical studies, in which each subject is monitored over a period of time [5, 6].

Classification of binary data [7], however, presents inherent challenges, primarily because any unseen sample to a classifier does not belong to the convex hull of the training data and therefore all predictions amount to extrapolation [8–10]. Quantifying the uncertainty of extrapolations stands out as a significant challenge, especially considering the presence of noise in data.

In supervised learning, noise refers to errors or inconsistencies in the data labeling [11, 12]. If the noise is significant in the training data, a data-driven model may learn noise-specific variations rather than underlying patterns generalizable to unseen data. This can lead to overfitting, where the model performs well on the training data but poorly on new data. Overfitting can occur when a model has too many parameters relative to the size of the training data. Several techniques have been introduced to prevent overfitting, such as regularization [13], cross-validation [14], and early stopping [15]. Such conventional techniques are commonly used to prevent excessive increases in classification loss on evaluation data through the training process [16]. However, when the evaluation data lies beyond the convex hull of the training data, the association between the loss functions of training and evaluation datasets loses clarity. To address this challenge, extrapolation-enhanced approaches are required, such as incorporating existing feature knowledge into learning within the framework of hybrid modeling.

The concept of hybrid mechanistic/data-driven modeling was developed in the early 1990 s to combine prior knowledge about the system of interest with data-driven modeling [17, 18]. Such methods are frequently used in the context of process and chemical engineering [19–22]. In a structured hybrid model (SHM) [23, 24], the prior knowledge about the system of interest serves as the structure of the information flow from input features to the outputs through different subsystems. The central idea of SHMs is to use structural knowledge to reduce the modeling complexity. As attested by the curse of dimensionality [25], the complexity of a black-box model increases exponentially with the dimension of its input. Purely data-driven models encounter high complexity as the mapping between input variables and outputs is modeled by a single black box that receives all variables in the modeling as its input. In contrast, an SHM conducts the information flow from input variables to outputs through several subsystems consisting of white boxes (known processes) and black boxes (unknown processes). Each black box of an SHM receives fewer input variables than the single black box in purely data-driven models. Due to the reduction in complexity compared with pure data-driven models, SHMs can significantly reduce the number of datasets needed to identify the model without sacrificing accuracy [26].

In this work, we utilized a prime example of SHMs, so-called functional networks (FNs) [27, 28], as a model class for the classification of binary data with prior knowledge on input features. FNs can be viewed as modular neural networks, where the structure of the links between the modules and the information flow from input variables to the output variable is pre-determined. Each module within the FN , henceforth referred to as a box, can serve as an independent data-driven model. The identification of an FN, i.e., learning the input–output (I/O) function of the FN, is then decomposed to the identification of the individual interior boxes.

**Fig. 1:**
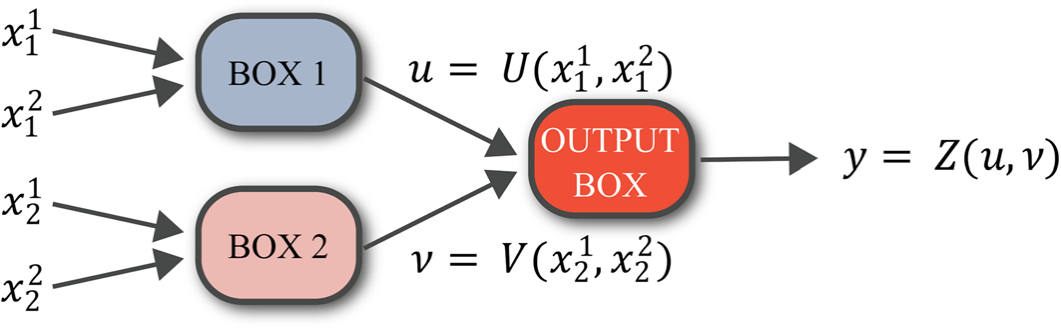
The visual representation of illustrative introduced in Eqs. (1–4)

Figure 1 shows a simple FN mapping input vector $\textbf{x} = [x_1^1, x_1^2, x_2^1, x_2^2]$ to output variable *y*. The subscript *i* indicates the input to box *i* while superscripts are ascending numbers enumerating the number of inputs to that box. In this example, the structure of the information flow from the input features $\textbf{x} \in \mathbb {R}^4$ to the output $y \in \mathbb {R}$ stems from assumed prior knowledge on the input features. This prior knowledge attests that the main process $\textrm{F}(\textbf{x}) = y$ can be decomposed into two sub-processes $\textrm{U}$ and $\textrm{V}$, and a complementary process $\textrm{Z}$ on the outputs of the sub-processes towards the final output of the main process:

1 $$\begin{aligned} y&= \textrm{F}(\textbf{x}) ,\quad \textbf{x} \in \mathbb {R}^4 \; ,\; y \in \mathbb {R}, \quad \textrm{F}: \mathbb {R}^4 \longmapsto \mathbb {R}, \end{aligned}$$

2 $$\begin{aligned} u&= \textrm{U}(x_1^1, x_1^2) , \quad u \in \mathbb {R} , \quad \textrm{U}: \mathbb {R}^2 \longmapsto \mathbb {R}, \end{aligned}$$

3 $$\begin{aligned} v&= \textrm{V}(x_2^1, x_2^2), \quad v \in \mathbb {R}, \quad \textrm{V}: \mathbb {R}^2 \longmapsto \mathbb {R}, \end{aligned}$$

4 $$\begin{aligned} y&= \textrm{Z}(u, v), \quad y \in \mathbb {R}, \quad \textrm{Z}: \mathbb {R}^2 \longmapsto \mathbb {R}. \end{aligned}$$

In this example, each sub-process separately performs computations on a subset of input features, here $\{x_1^1, x_1^2\}$ and $\{x_2^1, x_2^2\}$. In general, if there is no common feature between the subset of input features to the boxes of a two-layered FN, then the structure of the associated FN has a so-called tree structure.

The identification of FNs has been studied in detail in [23, 24] for tree-structured networks that map continuous input variables $\textbf{x} \in \mathbb {R}^n$ to continuous output variables in $y \in \mathbb {R}$. The proof of extrapolation used in [23, 24] is based on assumed densely distributed training data on low-dimensional subsets of $\mathbb {R}^n$, and monotonicity in the functions of subsystems. Towards the identification of tree-structured FNs that map randomly distributed binary input data $\textbf{x} \in \{0, 1\}^n$ to binary outputs $y \in \{0, 1\}$, a training strategy has been introduced in [26]. The training strategy uses graph-theoretic methods to analyze the data and identify the function of each box of an FN. However, the limitation of the strategy is to be relatively sensitive to noise in the data labeling.

In this work, in order to overcome the noise sensitivity observed in [26], we formulated the identification of each box of an FN as solving a maximum-cut (max-cut) problem. The max-cut problem is a well-known NP-hard combinatorial optimization problem and can be formulated as follows: given a graph $G = (V, E)$, find a partition of the vertices *V* into two sets $V_1$ and $V_2$ such that the sum of the weights of the edges connecting $V_1$ and $V_2$ is maximized. There are several algorithms that have been proposed to solve the max-cut problem, including spectral methods [29], randomized algorithms [30], and semi-definite programming [31]. However, most of these algorithms are only able to find approximate solutions, and there is still ongoing research to find more efficient and accurate methods to solve this problem. We chose to incorporate a max-cut problem into our learning strategy because maximizing the sum of weights associated with the cut necessitates excluding non-essential or weak connections between vertices. This selective exclusion can be utilized to filter out relatively infrequent noisy observations.

The introduced Python package in this work, named NoiseCut (standing for noise-tolerant classification of binary data using prior knowledge integration and max-cut solutions), not only exhibited remarkable robustness against noise but also showcased a capacity for generalization to non-tree structured FNs, a capability notably absent in [26].

The paper is structured as follows: The section “Implementation” presents the Python classes utilized in the NoiseCut package, accompanied by a code snippet outlining the complete workflow. In the section “Material and methods”, we first introduce the data utilized in this study, followed by an explanation of the derivation of the hybrid model and a mathematical description of the function identification employed in the learning strategy of NoiseCut. The section “Results” demonstrates the utility of NoiseCut through two use cases: noise-tolerant classification and classification with reduced training data. In the section “Discussion”, we delve into the noise-mitigation process within NoiseCut, addressing its interpretability and areas for future research. Finally, we conclude with a brief section “Conclusion” that highlights the limitations of our approach.

## Implementation

The NoiseCut package is implemented in Python, and its core functionality is organized into four main Python classes, each serving distinct roles: SampleGenerator, DataManipulator, NoiseCut, and Metric.

As the first step, data integration can be achieved through two approaches. Users can manually upload their data using the Pandas library [32], allowing for incorporation of their existing datasets. Alternatively, the SampleGenerator class can be implemented for the generation of synthetic data, providing a customized approach for experimentation and testing. For a detailed explanation of the synthetic data generation process, please refer to the supplementary information (Additional file 1), which covers cases where functions within the functional networks are either randomly assigned or manually specified.

Moving forward, the DataManipulator class adds noise to data by flipping binary labels of randomly selected samples. DataManipulator class also manages data partitioning into training and test sets. The NoiseCut class takes the provided training set and fits it into the hybrid model. This step implements the function identification of the FNs’ interior boxes by solving particular max-cut problems. The attribute “n_input_each_box” in the NoiseCut class defines the sole hyperparameter of the hybrid model as an array. Its length specifies the number of boxes in the first layer of the FN, while each element in the array denotes the number of inputs to each box in the first layer. The hyperparameter for each FN’s structure is predetermined and given by the assumed prior knowledge on the input features and their interactions. Finally, the Metric class calculates the evaluation metrics necessary for classification. This step allows for assessing the performance of the model effectively. For a comprehensive understanding of these Python classes within the package, please refer to the supplementary information (Additional file 1), which includes a practical usage example to assist in grasping the implementation details.

The code snippet below demonstrates the execution of the package. This code summarizes a complete workflow, starting with the generation of synthetic data, proceeding to the division of data into training and testing sets, and concluding with model fitting and result evaluation.
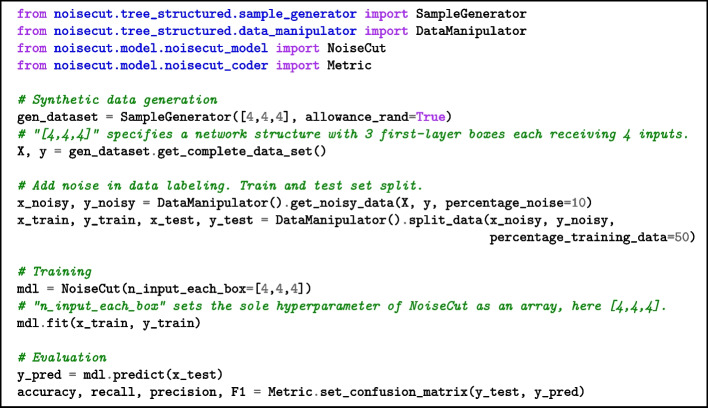


## Material and methods

### Data

**Fig. 2:**
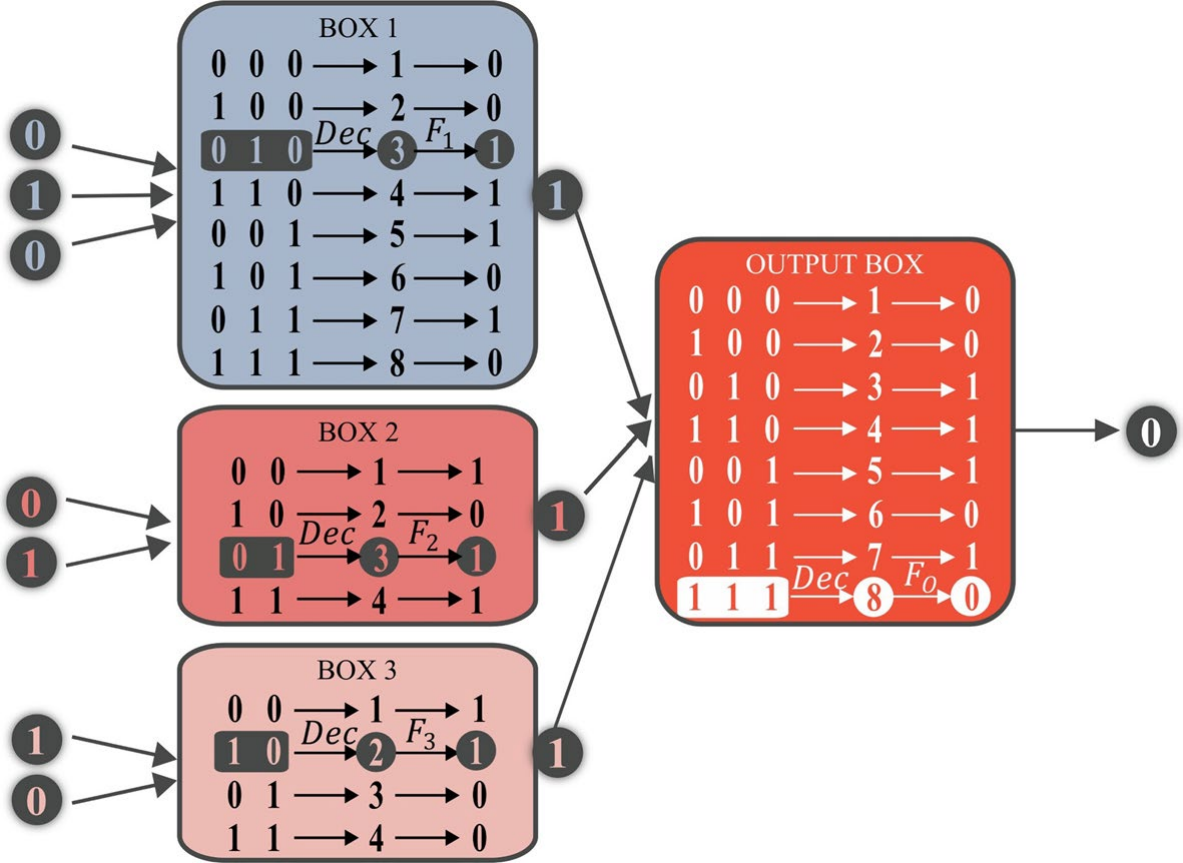
A schematic representation of the information flow from binary-represented input data to binary labels. This procedure has been used to generate the synthetic datasets

To comprehensively benchmark the noise resistance of NoiseCut against machine learning (ML) models, we employed 30 synthetically generated datasets. These datasets were generated with the assumption that prior knowledge about the features is available. This knowledge includes both the input features involved in the classification task and the structure of the information flow from these inputs to the output labels. The structure is defined by tree-structured networks, as illustrated in Fig. 2. While NoiseCut is compatible with real-world data, we opted for synthetic datasets in the benchmark to eliminate potential uncertainties related to prior knowledge of real-world features. This choice ensures a more controlled evaluation of NoiseCut’s noise resistance against ML models.

Each tree structure in the generated synthetic datasets consists of two layers of boxes, with a varying number of binary-represented input features $\textbf{x} \in \{0,1\}^d$, where *d* ranges from 8 to 12. The first layer comprises three boxes, followed by an output box in the second layer. The output labels are binary, denoted as $y \in \{0, 1\}$. To provide NoiseCut with prior knowledge, an array of length three is required, with each element representing the number of inputs to each box in the first layer. Each tree structure was randomly constructed so that each first-layer box operates on 2–6 separate input entries and forwards the partial results to the output box. A total of six different network structures were created for each input dimension, resulting in 30 tree structures in total.

Figure 2 illustrates an example of the labeling procedure in the synthetic datasets. We assumed a tree-structured network $\mathcal {F}: \{0,1\}^7 \longmapsto \{0,1\}$ mapping binary variables $\textbf{x}$ to binary labels *y*:

$$\begin{aligned} y = \mathcal {F}(X), \quad \textbf{x} \in \{0,1\}^7, \quad y \in \{0,1\}. \end{aligned}$$

In the network of Fig. 2, there are three first-layer boxes $\mathrm {F_1}: \{0,1\}^3 \longmapsto \{0,1\}$, $\mathrm {F_2}: \{0,1\}^2 \longmapsto \{0,1\}$, and $\mathrm {F_3}: \{0,1\}^2 \longmapsto \{0,1\}$ that separately perform computations on subsets of input features. Here are the I/O functions of the first-layer boxes in Fig. 2:

$$\begin{aligned} F_1: \begin{pmatrix} 0 &{} \quad 0 &{} \quad 0 \\ 1 &{} \quad 0 &{} \quad 0 \\ 0 &{} \quad 1 &{} \quad 0 \\ 1 &{} \quad 1 &{} \quad 0 \\ 0 &{} \quad 0 &{} \quad 1 \\ 1 &{} \quad 0 &{} \quad 1 \\ 0 &{} \quad 1 &{} \quad 1 \\ 1 &{} \quad 1 &{} \quad 1 \\ \end{pmatrix}&\longmapsto \begin{pmatrix} 0 \\ 0 \\ 1 \\ 1 \\ 1 \\ 0 \\ 1 \\ 0 \\ \end{pmatrix},&F_2: \begin{pmatrix} 0 &{} \quad 0 \\ 1 &{} \quad 0 \\ 0 &{} \quad 1 \\ 1 &{} \quad 1 \\ \end{pmatrix}&\longmapsto \begin{pmatrix} 1 \\ 0 \\ 1 \\ 1 \\ \end{pmatrix},&F_3: \begin{pmatrix} 0 &{} \quad 0 \\ 1 &{} \quad 0 \\ 0 &{} \quad 1 \\ 1 &{} \quad 1 \\ \end{pmatrix}&\longmapsto \begin{pmatrix} 1 \\ 1 \\ 0 \\ 0 \\ \end{pmatrix}. \end{aligned}$$

For instance, when we enter $\textbf{x}^\prime = [0, 1, 0, 0, 1, 1, 0]$ to the network, the three first-layer boxes return [1, 1, 1], which is then forwarded to the output box $\mathrm {F_O}: \{0,1\}^3 \longmapsto \{0,1\}$ with the following I/O function:

$$\begin{aligned} F_O: \begin{pmatrix} 0 &{} \quad 0 &{} \quad 0 \\ 1 &{} \quad 0 &{} \quad 0 \\ 0 &{} \quad 1 &{} \quad 0 \\ 1 &{} \quad 1 &{} \quad 0 \\ 0 &{} \quad 0 &{} \quad 1 \\ 1 &{} \quad 0 &{} \quad 1 \\ 0 &{} \quad 1 &{} \quad 1 \\ 1 &{} \quad 1 &{} \quad 1 \\ \end{pmatrix}&\longmapsto \begin{pmatrix} 0 \\ 0 \\ 1 \\ 1 \\ 1 \\ 0 \\ 1 \\ 0 \\ \end{pmatrix} \end{aligned}$$

Finally, the output box returns the generated label, here $y^\prime =0$, for the entered input $\textbf{x}^\prime$ to the network.

The generated synthetic datasets encompass a combination of balanced and imbalanced configurations, with the ratio of the two binary output labels $(y \in \{0,1\})$ varying between 0.1875:0.8125 and 0.8125:0.1875. This deliberate variation allows us to evaluate the performance of NoiseCut under different class distribution scenarios, ensuring robustness for both balanced and imbalanced datasets.

While our study exclusively focuses on binary-represented datasets, the application of NoiseCut can be extended to include categorical data through the use of one-hot encoding for each category. In the case of continuous features, a straightforward approach involves binning the range of feature values into discrete intervals. This aligns with the learning strategy used for binary or categorical data, but it is crucial to acknowledge that the classification task will incorporate uncertainty arising from the binning process.

### Model

NoiseCut employs a hybrid mechanistic/data-driven model designed for binary classification of binary-represented data. Stemming from prior knowledge of features (mechanistic modeling component), sets of binary input features are independently directed to distinct interior boxes within a tree-structured FN composed of nested functions. The learning strategy (data-driven modeling component) involves identifying the function of these interior boxes using a set of labeled training dataset.

NoiseCut primarily focuses on tree-structured FNs with two layers. The first layer consists of first-layer boxes, each operating on separated subsets of input features, while the second layer contains only an output box that processes the outputs of the first-layer boxes to produce the overall FN output. The first-layer boxes, assumed to have binary outputs, are employed for sub-computations related to the main classification task.

In alignment with the terminology introduced by some researchers [33, 34], the first-layer boxes can also be interpreted as weak classifiers. The primary contribution of this study lies in formulating the identification of individual first-layer boxes as the solution to specific max-cut problems. The I/O function of the output box, which can be regarded as a strong classifier [33, 34], is identified through a majority voting scheme.

**Fig. 3:**
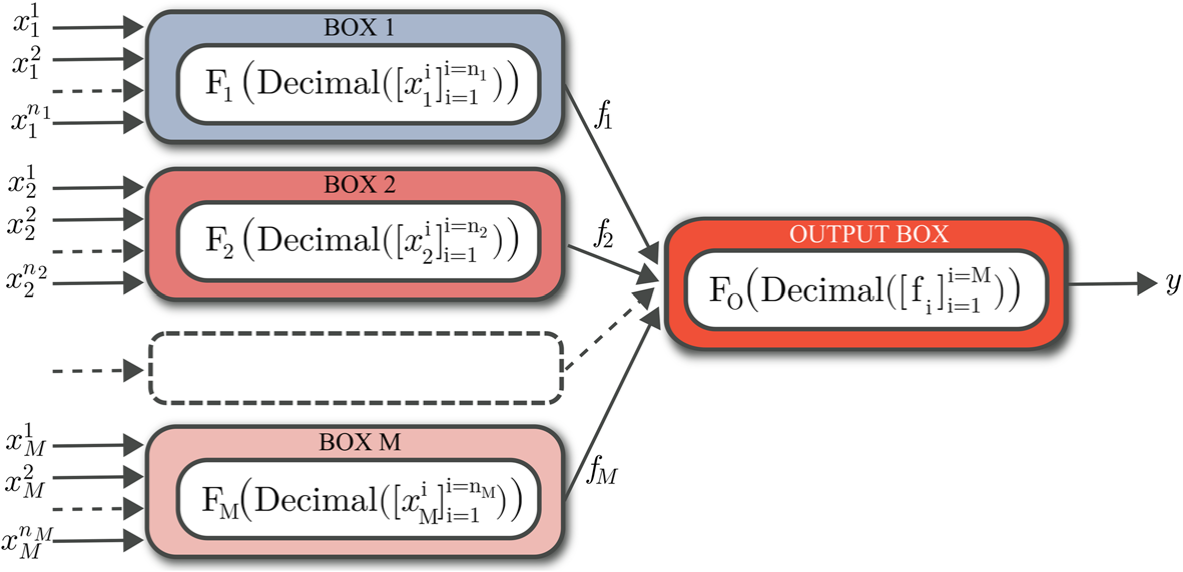
A tree-structured FN $\mathcal {F}: x \in \{0, 1\}^N \longmapsto y \in \{0, 1\}$, which maps binary-represented data to binary output. The FN has *M* first-layer boxes, operating on separate subsets of the input variables: $f_m = \textrm{F}_m(\text {Decimal}\Big ([x_m^i]_{i=1}^{i=n_m}\Big ))$. The output box in the second layer processes the outcomes of the first-layer boxes towards the overall output of the FN: $y = \textrm{F}_O(\text {Decimal}\Big ([f_i]_{i=1}^{i=M}\Big ))$

Consider the FN $\mathcal {F}: \{0, 1\}^N \longmapsto \{0, 1\}$ shown in Fig. 3. Let $\textbf{x} \in \{0, 1\}^N$ be an N-dimensional binary represented input vector to the network and $y \in \{0, 1\}$ be the associated output or label. The challenge is to use a given training set of *S* examples $\{(\textbf{x}_s, y_s) | s = 1, \ldots , S\}$ to deduce the I/O function of all *M* first-layer boxes and the I/O function of output box that accurately labels data points that are not in the training set.

Based on the structure of the FN in Fig. 3, N-dimensional binary input vector to the network can be decomposed into *M* vectors, $\big [ [x_1^1,\ldots , x_1^{n_1}],\ldots , [x_M^1,\ldots , x_M^{n_M}] \big ],$ which first-layer boxes separately perform computations on. Accordingly, $[x_m^i]_{i=1}^{i=n_m}$ is the subset of input features forwarded to the *m*th first-layer box, where $n_m$ is the size of the subset or the dimension of the binary input space of the *m*th first-layer box, $\sum _{m=1}^M n_m = N$, and each $x_m^i \in \{0, 1\}$.

Before feeding an $n_m$-dimensional binary variable to the *m*th first-layer box, we convert it to the associated decimal representation:

5 $$\begin{aligned} \text {Decimal}\Big ([x_m^i]_{i=1}^{i=n_m}\Big ) = 1 + \sum _{i=1}^{i=n_m} 2^{i-1} \times x_m^i. \end{aligned}$$

Therefore, an N-dimensional binary input vector $\textbf{x} \in \{0, 1\}^N$ to the FN on Fig. 3 can be represented as an *M*-dimensional vector $\mathcal {X} \in \mathbb {N}^M$:

6 $$\begin{aligned} \mathcal {X} = \Big [ \text {Decimal}\Big ([x_1^i]_{i=1}^{i=n_1}\Big ), \ldots , \text {Decimal}\Big ([x_M^i]_{i=1}^{i=n_M} \Big ) \Big ]. \end{aligned}$$

For example, the function $\textrm{F}_m$ of the *m*th first-layer box, receives a decimal value in $V_m = \{v_m^k\}_{k=1}^{k=2^{n_m}}$, where the value of $v_m^k$ is equal to *k*, and forwards a binary value $f_m$ to the output box:

7 $$\begin{aligned} f_m = \textrm{F}_m \Big (\text {Decimal}\Big ([x_m^i]_{i=1}^{i=n_m}\Big ) \Big ), \qquad x_m^i \;, f_m \in \{0, 1\}, \qquad \textrm{F}_m : V_m \longmapsto f_m, \end{aligned}$$

where $m \in \{1, 2,\ldots , M\}$. Then, the output box receives an M-dimensional binary variable $[f_i]_{i=1}^{i=M} \in \{0, 1\}^M$ from all *M* first-layer boxes. After converting it to a decimal value, which is in $V_O = \{v_M^k\}_{k=1}^{k=2^{M}}$, the function of the output box $\textrm{F}_O$ returns the predicted label:

8 $$\begin{aligned} y = \textrm{F}_O \left( \text {Decimal}\Big ([f_i]_{i=1}^{i=M}\Big ) \right) , \qquad f_i \;, y \in \{0, 1\}, \qquad \textrm{F}_O : V_O \longmapsto y. \end{aligned}$$

### Learning strategy

NoiseCut’s learning strategy utilizes graph-theoretic methods to analyze training data, mapping the function identification of individual first-layer boxes in a given FN to solving max-cut problems. For each first-layer box of the FN, a conflict graph *G*(*V*, *E*) is defined, enabling the use of graph-theoretic methods to deduce its I/O function.

In the conflict graph $G_m(V_m, E_m)$ of the *m*th first-layer box, $V_m$ represents the decimal values corresponding to the inputs of the box, and $E_m$ denotes an edge $v_m^k v_m^l$ with weight $w_{m}^{kl}$, indicating the dissimilarity between the function $F_m$ of the box for the associated vertices $v_m^k$ and $v_m^l$.

The primary goal of the learning strategy, preceding the function identification phase, is to determine how to ensure $\textrm{F}_m(v_m^k) \ne \textrm{F}_m(v_m^l)$ and how to establish edge weights $w_{m}^{kl}$ for a conflict graph. To tackle the former, let’s consider two input samples $\textbf{x}$ and $\textbf{x}^\prime$, both belonging to the FN depicted in Fig. 3, where the inputs to all first-layer boxes except the *m*th first-layer box remain identical:

9 $$\begin{aligned} \exists ! \; m \in \{1, \ldots , M\} \; \ni \; [x_m^i]_{i=1}^{i=n_m} \ne [{x^\prime }_m^i]_{i=1}^{i=n_m}. \end{aligned}$$

For $v_m^k = \text {Decimal}\Big ([x_m^i]_{i=1}^{i=n_m}\Big )$ and $v_m^l = \text {Decimal}\Big ([{x^\prime }_m^i]_{i=1}^{i=n_m}\Big )$, $\textrm{F}_m(v_m^k) \ne \textrm{F}_m(v_m^l)$ if and only if $\textbf{x}$ and $\textbf{x}^\prime$ yield different labels, $y_\textbf{x} \ne y_{\textbf{x}^\prime }$; please refer to the proof provided in the supplementary information (Additional file 1).

Next, we assign weights to conflict graph edges. In a conflict graph *G*(*V*, *E*), edges *E* can be given weights *W* to signify the strength or importance of the connection between connecting vertices. In this context, each edge indicates differing outputs for associated input vertices in the first-layer box. To establish these weights, all ${S \atopwithdelims ()2}$ pairs of input samples $\textbf{x}$ and $\textbf{x}^\prime$ in the training data $\{(\textbf{x}_s, y_s) | s = 1,\ldots , S\}$ are considered. If the selected pairs possess distinct labels and satisfy the condition in Eq. (9), the associated edge’s weight in $G_m(V_m, E_m)$ is incremented by one.

In the example of the network structure of Fig. 3, we define a $2^{n_m} \times 2^{n_m}$ weight matrix $W_m$ for the *m*th first-layer box by:

10 $$\begin{aligned} W_m = 0_{2^{n_m}, 2^{n_m}} + \sum _{\mathrm {all\;pairs\;(\textbf{x},\textbf{x}^\prime )}} |y_\textbf{x} - y_{\textbf{x}^\prime }| \times \prod _{i=1\;,\;i \ne m}^{i=M} \delta (\mathcal {X}[i] - \mathcal {X}^\prime [i]) \times \textbf{e}_{\mathcal {X}[i]} \textbf{e}_{\mathcal {X}^\prime [i]}^T , \end{aligned}$$

where $\mathcal {X}, \mathcal {X}^\prime \in \mathbb {N}^M$ are M-dimensional decimal representations of binary input vectors $\textbf{x}, \textbf{x}^\prime \in \{0, 1\}^N$ with labels $y_\textbf{x}, y_{\textbf{x}^\prime } \in \{0, 1\}$, $\delta$ is the Kronecker delta function, and $\textbf{e}_i$ are elements of the standard basis of vector space $\mathbb {R}^{2^{n_m}}$:

11 $$\begin{aligned} \textbf{e}_1 = [1, 0, 0, \ldots , 0]^T \;,\; \textbf{e}_2 = [0, 1, 0, \ldots , 0]^T \;,\; \ldots \;,\; \textbf{e}_{2^{n_m}} = [0, 0, 0, \ldots , 1]^T . \end{aligned}$$

After determining the weight matrices of all *M* conflict graphs based on the training data at hand, we identify the function of the first-layer boxes by partitioning the vertices of the conflict graphs into two sets. The max-cut problem is used to find the best partition of the vertices that maximizes the sum of the weights of the edges connecting the two sets [35]: Let binary variables $x_i$ for every vertex in a graph *G*(*V*, *E*) be such that $x_u = 1$ if $u \in V_1$ and $x_u = 0$ if $u\in V_2$, and $y_{uv}$ be a binary variable indicating whether edge *uv* is cut by the partition $(y_{uv} = 1)$ or not $(y_{uv} = 0)$. Then the mixed integer linear programming (MILP) formulation of the max-cut problem is given by:

12 $$\begin{aligned} \textrm{max} \; \sum _{v=1}^n \sum _{u=1}^{v-1} w_{uv} \; . \; y_{uv} \; ,&\end{aligned}$$

13 $$\begin{aligned} s.t. \; \; y_{uv} - x_u - x_v \le 0, \; \; \; \;&u,v = 1, 2, \ldots , n,\; \; u<v,\end{aligned}$$

14 $$\begin{aligned} y_{uv} + x_u + x_v \le 2, \; \; \; \;&u,v = 1, 2, \ldots , n,\; \; u<v,\end{aligned}$$

15 $$\begin{aligned} y_{uv} \in \{0,1\}, \; \; \; \;&u,v = 1, 2, \ldots , n,\; \; u<v,\end{aligned}$$

16 $$\begin{aligned} x_u \in \{0,1\}, \; \; \; \;&u = 1, 2, \ldots , n, \end{aligned}$$

where *n* is the number of vertices in *G*(*V*, *E*), and $w_{uv}=0$ if and only if there is no edge between vertices *u* and *v*. The solution to above mentioned max-cut problem for a conflict graph $G_m(V_m, E_m)$ with a weight matrix $W_m$ provides a function approximation for the *m*th first-layer box of the FN shown in Fig. 3; please refer to the proof provided in the supplementary information (Additional file 1).

NoiseCut employs an extension of the branch-and-bound (BB) algorithm, utilizing the CPLEX solver [36] to solve the MILP formulation of the specified max-cut problems for each first-layer box. To start, BB solves the “relaxed” problem, allowing $y_{uv} \in \{0, 1\}$ to take continuous values $\in [0, 1]$, providing a global lower bound on the objective function. If all the variables $y_{uv}$ have integer values (here 0 or 1), this solution becomes the global solution to the original problem. If there are non-integer values, BB branches by selecting one variable and creates two subproblems, fixing the variable to 0 in one and 1 in the other. If an integer solution is found in either subproblem, the associated objective value becomes an upper bound. The best upper bound is updated if a smaller one is discovered. BB proceeds iteratively by addressing non-integer variables, eliminating infeasible subproblems, and pruning subproblems in which the local lower bound exceeds the best upper bound, until all subproblems are either solved or eliminated. This systematic process guarantees finding an optimal solution in a finite number of iterations in MILP problems.

Lastly, NoiseCut identifies the I/O function $\textrm{F}_O: V_O \longmapsto y$ of the output box. As shown in Eq. (8), the output box receives the decimal representations of the outcomes of the first-layer boxes $[f_i]_{i=1}^{i=M}$ and assigns a binary label $y \in \{0, 1\}$ to each of them. In order to identify the I/O function $\textrm{F}_O$ of the output box, NoiseCut uses a majority voting scheme as follows: inputs to the output box are in $V_O = \{1, 2, 3,\ldots , 2^{M}\}$, and can be related to multiple input samples $(\textbf{x}_s, y_s)$ in the training data set $\{(\textbf{x}_s, y_s) | s = 1,\ldots , S\}$. For each element $v_O^k$ in $V_O$, the number of times that the associate sample $\textbf{x}_s$ in the train data set have labels $y_s$ equal 0 or 1 is counted. Then, the label with the most votes will be assigned as the outcome of the output box function for $v_O^k$.

## Results

To showcase the utility of NoiseCut, we present the results of two use cases involving the classification of binary data with prior knowledge of features. The first use case demonstrates noise tolerance in the classification of binary data, while the second one focuses on classification with reduced training data. To evaluate NoiseCut’s performance for these two use cases, we conduct benchmarking tests against various supervised ML algorithms, namely Deep Neural Networks (DNNs) [37], eXtreme Gradient Boosting (XGBoost) [38], Support Vector Machine (SVM) [39], and Random Forest (RF) [40]. The entire analysis can be explored through interactive Python notebooks, conveniently named “Noise-tolerant classification.ipynb” and “Classification with reduced training data.ipynb”, accessible at the following link: https://github.com/JRC-COMBINE/NoiseCut/tree/main/docs/notebooks.

### Noise-tolerant classification

In the initial use case, we assess the performance of NoiseCut in classifying binary data with noisy labels, comparing it to different ML algorithms. To assess performance, we performed five experiments on each of the 30 generated synthetic datasets. These experiments aimed to measure classification metrics on testing data across various noise intensities in data labeling, maintaining a consistent 70% training data size across all cases.

We employed grid-search cross-validation [41] as a hyperparameter tuning method for DNN, XGBoost, and RF models. Specifically, we utilized 5-fold stratified cross-validation on shuffled training data. The performance of the selected hyperparameters and trained models was then evaluated on a dedicated test set that was kept separate during the training process. To prevent overfitting, we applied the early stopping method with a tunable waiting time, which was optimized as a hyperparameter for all the ML models.

The results presented in Table 1 showcase the remarkable noise tolerance of NoiseCut, as evidenced by its consistently high accuracy, recall, precision, F1 score, and Area Under the Receiver Operating Characteristic Curve (AUC-ROC) values for noise intensities ranging from 0 to 10%. At a noise intensity of 0%, NoiseCut achieves perfect classification performance and excels at preserving high precision and recall. Even as noise intensifies to 10%, NoiseCut maintains a robust performance, achieving a classification accuracy of 0.887 ± 0.006, showcasing its effectiveness in handling overfitting.

In contrast, DNN, XGBoost, and SVM demonstrate a noticeable decline in performance with increasing noise intensity. While they still achieve remarkable results at lower noise levels (e.g., 0% noise with a classification accuracy of 0.993 ± 0.011 for DNN, 0.974 ± 0.008 for XGBoost, and 0.934 ± 0.009 for SVM), their performance reduces significantly under the influence of noise in data labeling (e.g., 10% noise with a classification accuracy of 0.807 ± 0.016 for DNN, 0.808 ± 0.008 for XGBoost, and 0.771 ± 0.009 for SVM). Notably, the performance of RF underperformed the others, even at 0% noise, with a classification accuracy of 0.883 ± 0.010.

**Table 1:** The median with 95% CI of classification metrics for NoiseCut, DNN, XGBoost, SVM, and RF on testing data across different noise intensities in data labeling. The training data size was 70% for all the experiments

**Noise Intensity**	**Model**	**Accuracy**	**Recall**	**Precision**	**F1 Score**	**AUC-ROC**
0%	NoiseCut	1.000 ± 0.000	1.000 ± 0.000	1.000 ± 0.000	1.000 ± 0.000	1.000 ± 0.000
	DNN	0.993 ± 0.011	0.998 ± 0.002	0.996 ± 0.006	0.993 ± 0.012	0.999 ± 0.001
	XGBoost	0.974 ± 0.008	0.983 ± 0.019	0.975 ± 0.012	0.975 ± 0.015	0.998 ± 0.004
	SVM	0.934 ± 0.009	0.941 ± 0.015	0.934 ± 0.014	0.929 ± 0.013	0.966 ± 0.009
	RF	0.883 ± 0.010	0.905 ± 0.028	0.880 ± 0.016	0.890 ± 0.021	0.949 ± 0.009
2.5%	NoiseCut	0.974 ± 0.001	0.980 ± 0.006	0.973 ± 0.002	0.975 ± 0.003	0.967 ± 0.003
	DNN	0.933 ± 0.015	0.949 ± 0.034	0.922 ± 0.022	0.934 ± 0.031	0.955 ± 0.012
	XGBoost	0.925 ± 0.008	0.941 ± 0.020	0.927 ± 0.012	0.931 ± 0.016	0.954 ± 0.006
	SVM	0.874 ± 0.009	0.885 ± 0.017	0.879 ± 0.015	0.892 ± 0.015	0.896 ± 0.009
	RF	0.857 ± 0.011	0.873 ± 0.030	0.857 ± 0.017	0.867 ± 0.022	0.908 ± 0.010
5%	NoiseCut	0.947 ± 0.004	0.957 ± 0.013	0.950 ± 0.007	0.951 ± 0.008	0.934 ± 0.004
	DNN	0.891 ± 0.016	0.883 ± 0.046	0.895 ± 0.036	0.892 ± 0.040	0.912 ± 0.019
	XGBoost	0.873 ± 0.008	0.890 ± 0.022	0.880 ± 0.013	0.891 ± 0.017	0.909 ± 0.007
	SVM	0.826 ± 0.011	0.857 ± 0.027	0.832 ± 0.018	0.838 ± 0.025	0.855 ± 0.019
	RF	0.818 ± 0.010	0.844 ± 0.031	0.820 ± 0.015	0.834 ± 0.023	0.865 ± 0.009
7.5%	NoiseCut	0.920 ± 0.005	0.924 ± 0.018	0.921 ± 0.011	0.917 ± 0.012	0.906 ± 0.006
	DNN	0.845 ± 0.019	0.833 ± 0.048	0.828 ± 0.028	0.843 ± 0.032	0.864 ± 0.019
	XGBoost	0.840 ± 0.009	0.868 ± 0.025	0.854 ± 0.014	0.858 ± 0.019	0.875 ± 0.008
	SVM	0.805 ± 0.010	0.819 ± 0.023	0.804 ± 0.016	0.811 ± 0.019	0.824 ± 0.010
	RF	0.798 ± 0.009	0.827 ± 0.033	0.803 ± 0.015	0.810 ± 0.024	0.828 ± 0.010
10%	NoiseCut	0.887 ± 0.006	0.891 ± 0.022	0.892 ± 0.014	0.887 ± 0.017	0.872 ± 0.007
	DNN	0.807 ± 0.016	0.830 ± 0.050	0.803 ± 0.034	0.824 ± 0.041	0.821 ± 0.018
	XGBoost	0.808 ± 0.008	0.831 ± 0.026	0.814 ± 0.014	0.824 ± 0.019	0.839 ± 0.009
	SVM	0.771 ± 0.009	0.786 ± 0.029	0.797 ± 0.016	0.784 ± 0.026	0.798 ± 0.011
	RF	0.771 ± 0.010	0.780 ± 0.034	0.872 ± 0.015	0.773 ± 0.024	0.799 ± 0.011

To quantitatively compare the performance of NoiseCut with other ML models, the Friedman test was employed, as it is recommended for comparing more than two classifiers over multiple datasets [42], which is the case in this study. In the Friedman test, each classifier is evaluated on the same dataset, and performance metrics are recorded. Subsequently, ranks are assigned to the classifiers based on their performance, and the average rank for each classifier is calculated across all performance metrics.

Table 2 presents the ranking of classifiers as determined by the Friedman test. The rankings are based on the average ranks of the algorithms across all testing datasets, considering noise intensities ranging from 0 to 10% and a training data size of 70% for all experiments. This analysis was conducted using the Statistical Tests for Algorithms Comparison (STAC) Python Library [43]. The findings consistently demonstrate NoiseCut as the best-performing method.

**Table 2:** Classifier rankings based on average ranks across testing datasets and noise intensities (0% to 10%) highlighting NoiseCut as the best-performing method

**Noise Intensity**	**Model**	**The Average Rank Across All Experiments**
0%	NoiseCut	1.540
	DNN	2.670
	XGBoost	2.930
	SVM	3.466
	RF	4.393
2.5%	NoiseCut	1.490
	DNN	2.340
	XGBoost	2.753
	SVM	3.416
	RF	5.000
5%	NoiseCut	1.506
	DNN	2.316
	XGBoost	2.730
	SVM	3.446
	RF	5.000
7.5%	NoiseCut	1.543
	DNN	2.473
	XGBoost	2.730
	SVM	3.253
	RF	5.000
10%	NoiseCut	1.553
	DNN	2.503
	XGBoost	2.676
	SVM	3.266
	RF	5.000

**Fig. 4:**
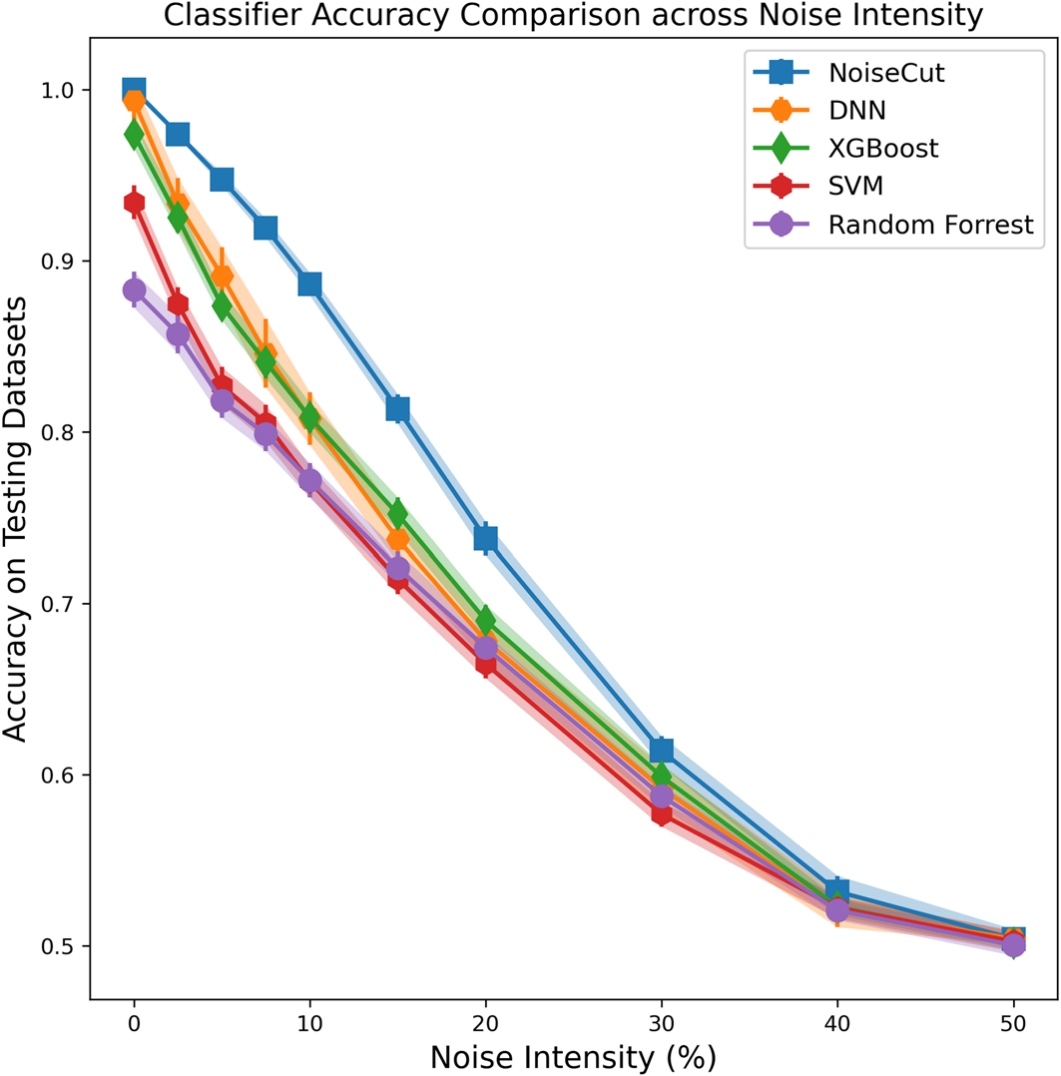
Classifier accuracy on testing datasets comparison of NoiseCut with various ML models for classifying binary data across the entire spectrum of noise intensities, with a consistent 70% training data size. NoiseCut outperforms the others as noise intensifies, demonstrating superior overfitting mitigation across varying levels of noise compared to the early stopping approach used by the other ML models

Figure 4 visualizes the comparison of classification accuracy between NoiseCut and various ML models across the entire range of noise intensities in data labeling, spanning from 0 to 50%, i.e., flipping binary labels of 0–50% of randomly selected samples. Although NoiseCut, DNN, and XGBoost demonstrate near-perfect performance in the absence of noise in data labeling, as the intensity of noise increases, NoiseCut outperforms the others. This underscores NoiseCut’s robustness in mitigating overfitting across varying levels of noise. It is noteworthy that, as the noise intensity reaches 50%, all models converge to a classification accuracy of around 50%, reflecting a scenario of random guessing.

### Classification with reduced training data

In our second use case, we aim to showcase NoiseCut’s effectiveness in classifying binary-represented data when training data is limited. For this purpose, we employed the 30 synthetic datasets and evaluated NoiseCut’s performance against DNN, XGBoost, and RF models.

**Fig. 5:**
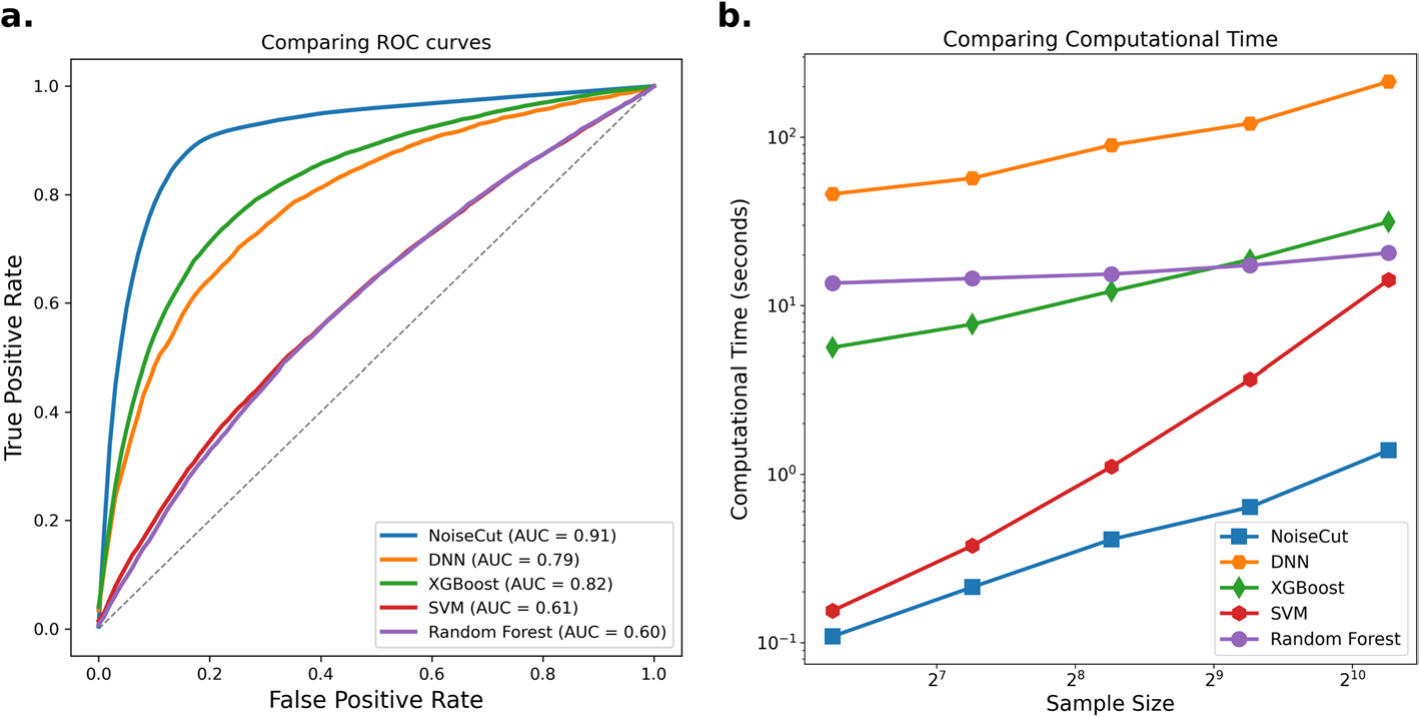
**a.** Comparison of ROC curves illustrating the classification performance of NoiseCut alongside other ML models on testing datasets. **b.** Comparison of computational time between NoiseCut and the other ML models across varying sample sizes. The evaluation is conducted with only 30% of the training data available and 5% noise intensity in the data labeling

In Fig. 5a, we compare the ROC curves of NoiseCut with those of other ML models, generated using testing datasets for the classification of synthetic datasets. This evaluation was conducted with only 30% of the training data available and a 5% noise intensity in the data labeling. NoiseCut demonstrates a superior performance with an AUC-ROC of approximately 0.91. In comparison, DNN achieves an AUC-ROC of 0.79, XGBoost follows with an AUC-ROC of around 0.82, SVM trails with an AUC-ROC of 0.61, and RF records an AUC-ROC of 0.60.

Figure 5b illustrates the comparison of computational time between NoiseCut and the other ML models across various sample sizes. The computation time accounts for model training and hyperparameter optimization for each dataset. The results suggest that the computational time of NoiseCut scales comparably with other models as the sample size increases within the datasets explored in this study.

These results indicate NoiseCut’s noteworthy performance in classifying binary data even when provided with limited training data. This advantage, highlighting the extrapolability of our method, primarily arises from the simplified structure of the FNs employed within NoiseCut. These FNs are designed to adapt to the synthetic data’s structure based on our assumed prior knowledge. For more in-depth technical discussion, please refer to the supplementary information (Additional file 1).

## Discussion

Although NoiseCut is compatible with real-world data, the examples and results showcased in this study utilize synthetic datasets. This choice is made due to the inherent noise present in real-world datasets, which constrains the systematic benchmarking of noise-free scenarios. Moreover, uncertainties in the prior knowledge of input features in real data introduce complexities that hinder a controlled comparison of NoiseCut’s noise-mitigating capabilities with other machine learning models. Nevertheless, validating NoiseCut’s efficacy on real-world datasets, particularly those with prior knowledge fitting a tree-structure FN with minimal uncertainty, remains an important pursuit, presenting an intriguing avenue for future research.

The noise-tolerant classification results highlight the synergy between NoiseCut’s hybrid structure and the utilization of defined max-cut problems for function identification. This combination proves to be more effective in preventing overfitting when compared to the early stopping technique used in various ML models, especially in the classification of binary data with corrupted labels. On one hand, the reduced complexity of NoiseCut’s hybrid structure, in contrast to a pure black-box model, serves as an inherent form of regularization. This can be construed as implementing a deliberate drop-out strategy, leveraging prior knowledge of input features to retain essential parameters for the model’s learning process while discarding non-essential ones.

On the other hand, NoiseCut excels in handling noisy labeling due to its alignment with the fundamental characteristics of max-cut problems. Specifically within the learning strategy of NoiseCut, a conflict graph is associated with each box in an FN. Pairs of samples from the training data are selected, and if they possess distinct labels and satisfy the condition outlined in Eq. (9), the weight of the corresponding edge in the conflict graph is incremented (Eq. 10 summarizes the whole weight association strategy for the example of the network structure of Fig. 3). This weight association indicates that connections between vertices reflect differences in the output of the box function for those respective inputs. Subsequently, solutions to the max-cut problem on the conflict graph are utilized to approximate the box function. This involves identifying the optimal partitioning of vertices into two distinct sets, with the objective of maximizing the total weights associated with the cut. Notably, this objective intentionally disregards non-essential or weak connections between vertices. By strategically excluding these non-significant connections, often arising from relatively infrequent configurations due to noisy labels, NoiseCut effectively achieves noise filtration.

In a final note, the tree structure of the FN employed in NoiseCut inherently enhances interpretability compared to complex ML models. In contrast to other tree-based models like XGBoost and Random Forest, where, despite the interpretability of individual trees, the ensemble nature may complicate the overall decision-making process, NoiseCut’s final predictions involve aggregating several pre-defined first-layer boxes using prior knowledge on features and their interactions. It remains clear within NoiseCut’s learning strategy which aspect or subset of input features each black box evaluates towards the final decision made by the output box.

## Conclusion

We present NoiseCut, an open-source software implemented in Python that facilitates structured hybrid modeling for binary data classification. Binary data and their classification are of eminent interest in medical and clinical applications. These applications often face challenges arising from inherent uncertainties in the data and limitations in the available training data resulting in effective noise in the data. By leveraging prior knowledge of features, NoiseCut promises reduced training data demand and enhanced robustness against noise in data labeling. Additionally, NoiseCut introduces a novel approach to avoid overfitting by integrating solutions to max-cut problems into the learning strategy. Max-cut solutions prioritize excluding non-essential or weak connections between vertices, filtering out infrequent noisy observations.

The learning strategy introduced by NoiseCut has certain limitations, such as the requirement for prior knowledge on input features, the ability to classify datasets only with binary labels, and the exponential increase in computation time required for exact solutions to max-cut problems. Tackling these challenges represents a potential area for future research. Utilizing Large Language Models could address the requirement for prior knowledge by capturing clinical or medical relationships in data features. Extending the application of NoiseCut to multi-class datasets reflects the challenges encountered when transitioning from solving a max-cut problem in a graph, which is known to be NP-hard, to addressing a multi-coloring graph problem, known to be NP-complete. The computational demands associated with solving max-cut problems can be addressed by leveraging the exponential computing power of quantum learning machines.

## Availability and requirements

Project name: NoiseCut

Project home page: https://pypi.org/project/noisecut

Operating system(s): Platform independent

Programming language: Python

Other requirements: Python($\ge$3.9), numpy, pandas, scipy, cplex, docplex

License: GNU GPL v3

Any restrictions to use by non-academics: none

### Supplementary Information

**Additional file 1**. The additional file delves into the details of the function identification strategy, synthetic data generation, and a usage example of NoiseCut.

## Data Availability

All the data generated or analyzed in this study along with NoiseCut’s source code, tutorials, and supplementary information files can be accessed at: https://github.com/JRC-COMBINE/NoiseCut.

## Abbreviations

AUC-ROC: Area under the receiver operating characteristic curve
CI: Confidence interval
DNNs: Deep neural networks
FN: Functional network
ICD: International classification of diseases
Max-cut: Maximum cut
MILP: Mixed integer linear programming
ML: Machine learning
NoiseCut: Noise-tolerant classification of binary data using prior knowledge integration and max-cut solutions
RF: Random Forest
SHM: Structured hybrid model
STAC: Statistical tests for algorithms comparison
SVM: Support vector machine
XGBoost: Extreme gradient boosting
