# Supplementary material for: Noisecut: a python package for noise-tolerant classification of binary data using prior knowledge integration and max-cut solutions
Source: BMC Bioinformatics. 2024 Apr 20;25:155. doi: 10.1186/s12859-024-05769-8 (PMC11031902; doi:10.1186/s12859-024-05769-8)
Supplement: Supplementary file 1 — Additional file 1. The additional file delves into the details of the function identification strategy, synthetic data generation, and a usage example of NoiseCut. [file 12859_2024_5769_MOESM1_ESM.pdf]

NoiseCut: A Python Package for Noise-Tolerant Classification of Binary Data using Prior Knowledge Integration and Max-Cut Solutions

Moein E. Samadi [1], Hedieh Mirzaieazar [1], Alexander Mitsos [2], and Andreas Schuppert [1]

1 Institute for Computational Biomedicine, RWTH Aachen University, Aachen, Germany

2 Process Systems Engineering (AVT.SVT), RWTH Aachen University, Aachen, Germany

February 28, 2024

Supplementary information

This supplementary file is structured into three distinct sections. Section 1 delves into the graph-theoretical description of the function identification strategy employed within NoiseCut. Section 2 focuses on synthetic data generation, providing users with a customized approach for experimentation and software testing. Section 3 provides a detailed usage example of NoiseCut, demonstrating its practical applications and how the tool can be effectively employed in real-world scenarios.

1 The Function Identification Strategy of NoiseCut

This section will delve into the details of the function identification strategy implemented within our Python package, NoiseCut. First, we will establish some existing definitions related to hybrid modeling and introduce the concept of functional networks (FNs) as a model class used in NoiseCut for the classification of binary data with prior knowledge on features. Subsequently, by providing some original definitions, we will unveil the graph-theoretic formulation of the function identification strategy of NoiseCut for an FN $\mathcal{F} : \{0,1\}^N \longmapsto \{0,1\}$, which maps binary represented data to binary output. Overall, this supplementary file aims to introduce a learning strategy designed to address the following problem:

Problem 1. *A binary classification task using binary data. Stemming from prior knowledge on features, the set of binary features is separately forwarded to different interior black boxes of an FN comprised of nested functions. The task is to deduce the function of interior black boxes (that are assumed to be binary functions) using a set of labeled training data.*

1.1 Preliminaries

Hybrid mechanistic/data-driven modeling is an approach that integrates prior mechanistic knowledge with the utilization of data-driven learning techniques. In the context of process and chemical engineering, hybrid models are also known as either semi-parametric models [1,2] or gray-box models [3]. These models combine parametric parts, called white boxes, with non-parametric parts, called black boxes. While parametric parts are based on mechanistic knowledge about the system, non-parametric parts are exclusively derived from data.

Definition 1 (Complexity of a black box)**.** *The complexity of a black box is defined as the minimal number of evaluations of solution candidates required to determine the input-output (I/O) function of the black box.*

Definition 2 (Structured hybrid modeling)**.** *In a structured hybrid model (SHM), the prior knowledge about the system of interest serves as the structure of the information flow from input variables to outputs through different subsystems. Moreover, the sum of the complexities of the black-box subsystems within the SHM should not exceed the complexity of a single black box mapping the input variables to outputs.*

Definition 3 (Convex set)**.** *A set* $\boldsymbol{X}$ *is convex if, for all points* $\boldsymbol{x}_1, \boldsymbol{x}_2 \in \boldsymbol{X}$ *and for all values of* $\alpha \in [0, 1]$*, the point* $\alpha \boldsymbol{x}_1 + (1 - \alpha)\boldsymbol{x}_2 \in \boldsymbol{X}$.

Definition 4 (Convex hull)**.** *The convex hull of a set of data points* $\boldsymbol{X} \triangleq \{\boldsymbol{x}_1, ..., \boldsymbol{x}_N\}$ *is the smallest convex set containing all points in* $\boldsymbol{X}$.

The dimension of the convex hull of a set of data points is defined as the number of attributes or the dimension d of the data points. As a consequence of the curse of dimensionality, the volume of the convex hull of a set of d-dimensional data points scales by $\frac{1}{d!}$ to the d-dimensional data space of the data points [4]. In machine learning, the convex hull of the given training data specifies the interpolation domain within which purely data-driven methods can make reliable predictions.

Definition 5 (Extrapolation)**.** *Extrapolation refers to predictions for a data point* $\boldsymbol{x}$ *when it lies outside the convex hull of the training data set* $\{\boldsymbol{x}_1, ..., \boldsymbol{x}_N\}$.

One of the key advantages of SHMs over pure data-driven models is the ability to extrapolate. The extrapolability of SHMs is the direct consequence of applying the mechanistic part or white boxes of the modeling to domains that are outside the convex hull of training data [5,6].

Containing black-box subsystems alone, an SHM is still able to extrapolate. In this case, the union of the convex hulls of the subsystems for the given training data determines the domain in which the SHM can make accurate predictions. As each black box of an SHM receives fewer input variables than the dimension of the training data points, the volume of the union of the convex hulls of the SHM subsystems is greater than the convex hull of the given training data. In this way, the extrapolation domain of an SHM containing only black-box subsystems derives from subtracting the volume of the union of the convex hulls of the SHM subsystems from the convex hull of the training data (or the interpolation domain). In general, the extrapolation range of SHMs is influenced by factors such as the number of subsystems alongside the maximum number of input variables among them, and the presence of noises and biases in the training data.

The advantages of SHMs are well-established and are based on the availability of densely distributed training data on low-dimensional sub-manifolds of data spaces with continuous variables in $\mathbb{R}^n$ [5,6]. This property limits applications of hybrid modeling to cases for which highly correlated data distributions are available around low-dimensional manifolds within the input data space, such as in process engineering. In contrast to such controlled systems, observational data collected through scientific experiments or monitoring of natural phenomena mostly reflect uncontrolled systems in which the data distribution is not squeezed around low-dimensional manifolds. Moreover, observational data are often discrete or even binary, prohibiting the concepts of proof of extrapolation used in continuous data structures.

A training strategy is introduced in [7] for the identification of FNs performing binary classification tasks, where training data points are randomly distributed within binary data spaces. The training

strategy showed reduced data demand compared to pure data-driven models, and robustness in binary classification performances against increased dimensionality of the input data. However, adding noise to data notably affects the classification efficiency restricting applications of the training strategy in [7] on cases with clean data.

In NoiseCut, in order to overcome the noise sensitivity observed in [7], we formulated the binary function approximation of the interior black boxes by graph partitioning. In general, a graph $G(V, E)$ consists of a set V of vertices and a set E of pairs of vertices, called edges. We say two vertices $u \in V$ and $v \in V$ are adjacent if there is an edge $uv \in E$ between them. The basic idea of binary function approximation by graph partitioning is to represent the inputs of the binary function as vertices V in a graph, and the output of the function as the partition of the vertices into two sets V_1 and V_2. Stemmed from given training data, the edges $uv \in E$ of the graph are assigned weights w_{uv} based on how well they disagree with the function's output. Based on the edge weights, the maximum-cut *(max-cut)* problem is then used to deduce the best separation of the vertices into two sets, which represent the two possible outputs of the binary function.

1.2 Model

In this work, we utilized a prime example of SHMs, so-called functional networks, as a model class for the classification of binary data with prior knowledge on input features.

Definition 6 (Functional network)**.** *Functional networks (FNs) are networks composed of nested functions, each representing a subsystem of the overall system. Information about how a system can be decomposed into several subsystems is the prior knowledge that, we assumed, is given.*

Definition 7 (Tree structure)**.** *If there is no common feature between the subset of input features to the black boxes of an FN, then the structure of the associated FN has a so-called tree structure.*

Without loss of generality, we focused our study on tree-structured FNs composed of two layers. The first layer contains first-layer boxes, and the second layer only contains an output box.

Definition 8 (First-layer box)**.** *The first layer of an FN with a tree structure consists of independent black-box modules, called first-layer boxes, operating on separated subsets of input features.*

Assumption 1. *The first-layer boxes were assumed to have binary outputs and were used to perform sub-computations of the main classification task.*

Based on the terminology of some researchers [8, 9], the first-layer boxes could also be considered as weak classifiers. The main contribution of this work is to formulate the identification of the individual first-layer boxes to the solving of particular max-cut problems.

Definition 9 (Output box)**.** *The second layer of a two-layered FN uses a black-box module called the output box to process the outputs of the first-layer boxes towards the overall output of the FN.*

The I/O function of the output box, which can also be considered as a strong classifier [8, 9], will be identified by a majority voting scheme.

Consider the FN $\mathcal{F} : \{0, 1\}^N \longmapsto \{0, 1\}$ shown in Figrue 1. Let $\mathbf{x} \in \{0, 1\}^N$ be an N-dimensional binary represented input vector to the network and $y \in \{0, 1\}$ be the associated output or label. The challenge is to use a given training set of S examples $\{(\mathbf{x}_s, y_s)|s = 1, ..., S\}$ to deduce the I/O function of all M first-layer boxes and the I/O function of output box that accurately labels data points that are not in the training set.

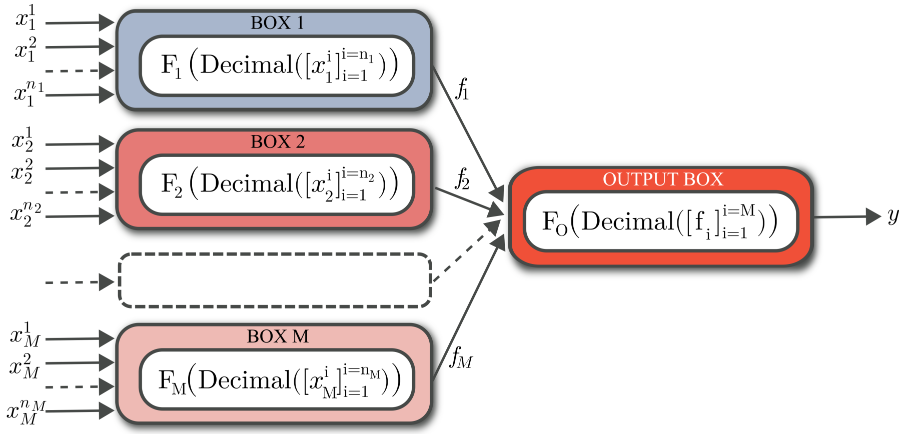

Figure 1: A tree-structured FN $\mathcal{F} : x \in \{0,1\}^N \longmapsto y \in \{0,1\}$, which maps binary represented data to binary output. The FN has M first-layer boxes, operating on separate subsets of the input variables: $f_m = \mathrm{F}_m(\mathrm{Decimal}\Big([x_m^i]_{i=1}^{i=n_m}\Big))$. The output box in the second layer processes the outcomes of the first-layer boxes towards the overall output of the FN: $y = \mathrm{F}_O(\mathrm{Decimal}\Big([f_i]_{i=1}^{i=M}\Big))$.

Based on the structure of the FN in Figure 1, N-dimensional binary input vector to the network can be decomposed into M vectors, which first-layer boxes separately perform computations on:

$$\big[[x_1^1, ..., x_1^{n_1}], ..., [x_M^1, ..., x_M^{n_M}]\big]. \tag{1.1}$$

Accordingly, $[x_m^i]_{i=1}^{i=n_m}$ is the subset of input features forwarded to the mth first-layer box, where n_m is the size of the subset or the dimension of the binary input space of the mth first-layer box, $\sum_{m=1}^{M} n_m = N$, and each $x_m^i \in \{0,1\}$.

Before feeding an n_m-dimensional binary variable to the mth first-layer box, we convert it to the associated decimal representation:

$$\mathrm{Decimal}\Big([x_m^i]_{i=1}^{i=n_m}\Big) = 1 + \sum_{i=1}^{i=n_m} 2^{i-1} \times x_m^i. \tag{1.2}$$

Therefore, an N-dimensional binary input vector $\mathbf{x} \in \{0,1\}^N$ to the FN on Figure 1 can be represented as an M-dimensional vector $\mathcal{X} \in \mathbb{N}^M$:

$$\mathcal{X} = \Big[\mathrm{Decimal}\Big([x_1^i]_{i=1}^{i=n_1}\Big), ..., \mathrm{Decimal}\Big([x_M^i]_{i=1}^{i=n_M}\Big)\Big]. \tag{1.3}$$

Definition 10 (Decimal subset)**.** *The decimal subset* $V_m = \{v_m^k\}_{k=1}^{k=2^{n_m}}$ *of the* m^{th} *first-layer box, where the value of* v_m^k *is equal to* k*, is defined as a set that holds the decimal representations of all* 2^{n_m} *binary configurations of* $[x_m^i]_{i=1}^{i=n_m}$*. In other words,* $V_m = \{v_m^1, v_m^2, v_m^3, ..., v_m^{2^{n_m}}\} = \{1, 2, 3, ..., 2^{n_m}\}$.

For example, the function F_m of the mth first-layer box, receives a decimal value in V_m and forwards a binary value f_m to the output box:

$$f_m = \mathrm{F}_m(\mathrm{Decimal}\Big([x_m^i]_{i=1}^{i=n_m}\Big)), \qquad x_m^i\,, f_m \in \{0,1\}, \qquad \mathrm{F}_m : V_m \longmapsto f_m, \tag{1.4}$$

where $m \in \{1, 2, ..., M\}$. Then, the output box receives an M-dimensional binary variable $\{0,1\}^M$ from all M first-layer boxes. After converting it to a decimal value, which is in the decimal subset

$V_O = \{1, 2, 3, ..., 2^M\}$ of the output box, the function of the output box F_O returns the predicted label:

$$y = \mathrm{F}_O(\mathrm{Decimal}\Big([f_i]_{i=1}^{i=M}\Big)), \qquad f_i\ , y \in \{0, 1\}, \qquad \mathrm{F}_O : V_O \longmapsto y. \tag{1.5}$$

1.3 Graph-theoretic Formulation of the Function Identification

The focus of our study is to use graph-theoretic methods to analyze a given training data and map the identification of the individual interior black-box modules of a given FN to the solving of max-cut problems. Accordingly, we defined a characteristic graph $G(V, E)$ for each first-layer box of the FN at hand in order to use graph-theoretic methods to deduce its I/O function.

Definition 11 (Characteristic graph)**.** *The characteristic graph $G_m(V_m, E_m)$ of the mth first-layer box is defined as follows: V_m is the decimal subset of the mth first-layer box and an edge $v_m^k v_m^l$ in E_m with the weight of w_m^{kl} represents the dissimilarity between the function F_m of the mth first-layer box for the associated vertices v_m^k and v_m^l.*

The main questions that need to be answered here are: "How to realize $\mathrm{F}_m(v_m^k) \neq \mathrm{F}_m(v_m^l)$ and how to set edge weights w_m^{kl} of a characteristic graph?"

Lemma 1. *For the FN of Figure 1, consider two input samples $\mathbf{x}, \mathbf{x}' \in \{0, 1\}^N$, for which the inputs to all first-layer boxes except for the mth first-layer box are identical:*

$$\exists!\ m \in \{1, ..., M\}\ \ni\ [x_m^i]_{i=1}^{i=n_m} \neq [x'^i_m]_{i=1}^{i=n_m}. \tag{1.6}$$

For $v_m^k = Decimal\Big([x_m^i]_{i=1}^{i=n_m}\Big)$ and $v_m^l = Decimal\Big([x'^i_m]_{i=1}^{i=n_m}\Big)$, $\mathrm{F}_m(v_m^k) \neq \mathrm{F}_m(v_m^l)$ iff $\mathbf{x}$ and $\mathbf{x}'$ have different labels $y_{\mathbf{x}} \neq y_{\mathbf{x}'}$.

Proof. According to Assumption 1 and Equations 1.4 and 1.5, $\mathrm{F}_m(v_m^k)$ and $\mathrm{F}_m(v_m^l)$ return either 0 or 1, and alongside the outputs of the other $M - 1$ first-layer boxes will be forwarded to the output box for the final computation towards the labels.

Based on the mathematical definition, a function has a unique output for a given input. Therefore, if for two input samples $\mathbf{x}$ and $\mathbf{x}'$ the inputs to all first-layer boxes except for the mth first-layer box are identical, then the related outputs of the first-layer boxes must be the same. Having different outputs for the output box, e.i., $y_{\mathbf{x}} \neq y_{\mathbf{x}'}$, the related inputs to the output box must be different. This difference can only be associated with the output of the mth first-layer box: $\mathrm{F}_m(v_m^k) \neq \mathrm{F}_m(v_m^l)$, because the outputs of the other $M - 1$ first-layer boxes must be the same. □

In the following, we briefly explain the procedure of assigning weights to the edges of characteristic graphs. The edges E in a graph $G(V, E)$ can be assigned weights W to show the importance or strength of the connection between the two vertices that the edge connects. As discussed above, an edge in a characteristic graph shows that the outputs of the first-layer box are different for the associated vertices to be the inputs. In Lemma 1, we only used a pair of input samples and analyzed them to draw an edge between two vertices of a characteristic graph. But this can also be done by selecting all $\binom{S}{2}$ pairs of input samples $\mathbf{x}$ and $\mathbf{x}'$ in given training data $\{(\mathbf{x}_s, y_s) | s = 1, ..., S\}$. If the selected pairs have different labels and meet the condition 1.6, then the weight of the associated edge in $G_m(V_m, E_m)$ will be increased by one.

Definition 12 (Weight matrix)**.** *In the example of the network structure of Figure 1, we define a* $2^{n_m} \times 2^{n_m}$ *weight matrix* W_m *for the* m^{th} *first-layer box by:*

$$W_m = 0_{2^{n_m},2^{n_m}} + \sum_{\text{all pairs } (\mathbf{x},\mathbf{x}')} |y_{\mathbf{x}} - y_{\mathbf{x}'}| \times \prod_{i=1\ ,\ i\neq m}^{i=M} \delta(\mathcal{X}[i] - \mathcal{X}'[i]) \times \mathbf{e}_{\mathcal{X}[i]}\mathbf{e}^T_{\mathcal{X}'[i]}, \tag{1.7}$$

where $\mathcal{X}, \mathcal{X}' \in \mathbb{N}^M$ *are* M*-dimensional decimal representations of binary input vectors* $\mathbf{x}, \mathbf{x}' \in \{0,1\}^N$ *with labels* $y_{\mathbf{x}}, y_{\mathbf{x}'} \in \{0,1\}$*,* δ *is the Kronecker delta function, and* $\mathbf{e}_i$ *are elements of the standard basis of vector space* $\mathbb{R}^{2^{n_m}}$*:*

$$\mathbf{e}_1 = [1,0,0,...,0]^T\ ,\ \mathbf{e}_2 = [0,1,0,...,0]^T\ ,\ ...\ ,\ \mathbf{e}_{2^{n_m}} = [0,0,0,...,1]^T. \tag{1.8}$$

After determining the weight matrices of all M characteristic graphs based on the training data at hand, we identify the function of the first-layer boxes by partitioning the vertices of the characteristic graphs into two sets. The max-cut problem is used to find the best partition of the vertices that maximizes the sum of the weights of the edges connecting the two sets [10].

Problem 2. *Let binary variables* x_i *for every vertex in a graph* $G(V,E)$ *be such that* $x_u = 1$ *if* $u \in V_1$ *and* $x_u = 0$ *if* $u \in V_2$*, and* y_{uv} *be a binary variable indicating whether edge uv is cut by the partition* $(y_{uv} = 1)$ *or not* $(y_{uv} = 0)$*. Then the mixed integer linear programming (MILP) formulation of the max-cut problem is given by:*

$$\max \sum_{v=1}^{n}\sum_{u=1}^{v-1} w_{uv} \cdot y_{uv}\ , \tag{1.9}$$

$$s.t.\ \ y_{uv} - x_u - x_v \leq 0, \quad u,v = 1,2,...,n, \quad u < v, \tag{1.10}$$

$$y_{uv} + x_u + x_v \leq 2, \quad u,v = 1,2,...,n, \quad u < v, \tag{1.11}$$

$$y_{uv} \in \{0,1\}, \quad u,v = 1,2,...,n, \quad u < v, \tag{1.12}$$

$$x_u \in \{0,1\}, \quad u = 1,2,...,n, \tag{1.13}$$

where n *is the number of vertices in* $G(V,E)$*, and* $w_{uv} = 0$ *iff there is no edge between vertices* u *and* v*.*

Extension of the branch and bound (BB) algorithm is commonly used for solving MILP problems [11], such as Problem 2. To start, BB solves the "relaxed" problem, allowing $y_{uv} \in \{0,1\}$ to take continuous values $\in [0,1]$, providing a global lower bound on the objective function. If all the variables y_{uv} have integer values (here 0 or 1), this solution becomes the global solution to the original problem. If there are non-integer values, BB branches by selecting one variable and creates two subproblems, fixing the variable to 0 in one and 1 in the other. If an integer solution is found in either subproblem, the associated objective value becomes an upper bound. The best upper bound is updated if a smaller one is discovered. BB proceeds iteratively by addressing non-integer variables, eliminating infeasible subproblems, and pruning subproblems in which the local lower bound exceeds the best upper bound, until all subproblems are either solved or eliminated. This systematic process guarantees finding an optimal solution in a finite number of iterations in MILP problems.

Theorem 1. *The solution to Problem 2 for a characteristic graph* $G_m(V_m, E_m)$ *with a weight matrix* W_m *provides a function approximation for the* m^{th} *first-layer box of the FN shown in Figure 1.*

Proof. Consider the function $F_m : V_m \mapsto f_m$, where V_m represents the vertices in the characteristic graph $G_m(V_m, E_m)$ and $f_m \in \{0,1\}$. F_m acts as a weak classifier discerning between samples $\mathbf{x}, \mathbf{x'} \in \{0,1\}^N$ with distinct labels $y_{\mathbf{x}} \neq y_{\mathbf{x'}}$ within the input space of the mth first-layer box in the FN of Figure 1.

The solution to Problem 2 seeks to partition V_m into V_{m_1} and V_{m_2} to maximize the sum of weights connecting V_{m_1} and V_{m_2}. Let y_{uv} be the binary variable indicating whether edge uv is cut by the partition ($y_{uv} = 1$) or not ($y_{uv} = 0$). The solution to Problem 2 for $G_m(V_m, E_m)$ with weight matrix W_m, defined in Definition 12, separates vertices corresponding to distinct labels $y_{\mathbf{x}} \neq y_{\mathbf{x'}}$ according to:

$$y_{uv} - F_m(u) - F_m(v) \leq 0, \quad \forall u, v \in V_m,\ u < v, \tag{1.14}$$

$$y_{uv} + F_m(u) + F_m(v) \leq 2, \quad \forall u, v \in V_m,\ u < v. \tag{1.15}$$

The partition induced by the max-cut solution defines the function F_m as follows:

$$F_m(v_m^k) \neq F_m(v_m^l), \quad \forall v_m^k \in V_{m_1}, v_m^l \in V_{m_2}. \tag{1.16}$$

Therefore, the solution to Problem 2 for $G_m(V_m, E_m)$ with weight matrix W_m provides a partitioning based on the training data, resulting in a function approximation F_m that discriminates between samples $\mathbf{x}, \mathbf{x'} \in \{0,1\}^N$ with distinct labels $y_{\mathbf{x}} \neq y_{\mathbf{x'}}$. □

Lastly, NoiseCut identifies the I/O function $\mathrm{F}_O : V_O \longmapsto y$ of the output box. As shown in Equation 1.5, the output box receives the decimal representations of the outcomes of the first-layer boxes $[f_i]_{i=1}^{i=M}$ and assigns a binary label $y \in \{0,1\}$ to each of them. In order to identify the I/O function F_O of the output box, NoiseCut uses a majority voting scheme as follows: inputs to the output box are in $V_O = \{1, 2, 3, ..., 2^M\}$, and can be related to multiple input samples $(\mathbf{x}_s, y_s)$ in the training data set $\{(\mathbf{x}_s, y_s) | s = 1, ..., S\}$. For each element v_O^k in V_O, the number of times that the associate sample $\mathbf{x}_s$ in the train data set have labels y_s equal 0 or 1 is counted. Then, the label with the most votes will be assigned as the outcome of the output box function for v_O^k.

The majority voting scheme for identifying the output box raises an important question: "What if there is no sample in the given training data that associates to an element v_O^k in V_O?" To answer this question, we first define an extension of the notion of a database from Refs. [5, 6] for binary data spaces.

Definition 13 (Binary database)**.** *For a FN $\mathcal{F} : \{0,1\}^N \longmapsto \{0,1\}$, a binary database $\mathcal{P}^N$ is defined as a set of data samples $\mathcal{P}^N \subset \{0,1\}^N$ with the size of the decimal subset V_O of the output box in the FN, where each element of $\mathcal{P}^N$ associates to one and only one element in V_O.*

The binary database of the FN in Figure 1 is a non-unique set $\mathcal{P}^N$ of 2^M N-dimensional binary data samples. Each data sample in $\mathcal{P}^N$ is associated to an element of the decimal subset $V_O = \{1, 2, 3, ..., 2^M\}$ of the output box.

In order to enable NoiseCut to extrapolate to the entire unseen data in $\{0,1\}^N$, the given training data should cover a binary database $\mathcal{P}^N \subset \{(\mathbf{x}_s, y_s) | s = 1, ..., S\}$. So, if there is no sample in the given training data set for identifying $\mathrm{F}_O(v_O^k)$, the I/O function identification of the output box

remains incomplete. Therefore, the FN cannot make faithful predictions for all unseen data, and their label determination is dependent on knowing $\mathrm{F}_O(v_O^k)$.

In this way, and in the case of having a training data set at hand that doesn't cover a binary database of the FN, our function identification method clarifies for which set of unseen data the FN can or cannot make faithful predictions. Therefore, our method somehow suggests a condition for adding new data samples – associated with missing elements in V_O – to the training data that have the most positive impact on the extrapolation range of the model.

1.4 Discussion

As outlined in Theorem 1, NoiseCut adopts a strategy for identifying the functions of the interior black boxes of an FN $\mathcal{F} : \{0,1\}^N \longmapsto \{0,1\}$ by tackling heuristic-defined max-cut problems. Based on the results shown in Table 1 of the main text, this strategy showcases better resilience to noise when compared to XGBoost. Specifically, NoiseCut proves to be more effective than the early stopping technique in mitigating overfitting in the classification of binary data.

The reason why NoiseCut performs well even when dealing with noisy labeling is because it aligns with the fundamental characteristics of max-cut problems. Specifically, the max-cut of a graph is designed to identify the optimal separation of vertices into two distinct sets, with the goal of maximizing the sum of weights associated with the cut. Achieving this objective necessitates the deliberate exclusion of non-essential or weak connections between vertices. These connections typically arise from relatively infrequent configurations brought about by the presence of noisy labels. This strategic disregard for non-significant connections is a key factor contributing to NoiseCut's effectiveness in handling noisy data.

2 Synthetic Data Generation

The presented guidelines in this section can be practically implemented using the `Generation of synthetic data.ipynb` notebook in the package documentation accessible on https://github.com/JRC-COMBINE/NoiseCut/blob/main/docs/notebooks/Generation_of_synthetic_data.ipynb.

2.1 Generating tree-structured data through randomly assigned functions

One can generate tree-structured synthetic data featuring an arbitrary number of first-layer boxes and an output box by using the NoiseCut package. The functionality of each box can be assigned randomly or manually specified.

For the generation of a tree-structured synthetic data set featuring interior boxes with randomly assigned functions, one can seamlessly employ the `SampleGenerator` class.

To instantiate an object of this class, you need to input an array which indicates the number of input features to each first-layer box. The first element of the array represents the number of input features to the first box, the second element represents the number of input features to the second box, and the rest follows the same. The length of the array is also an indicator of the number of first-layer boxes, which is `3` in the below example. If you set `allowance_rand=True`, all the functions are set randomly when the object is instantiated.

```
[1]: from noisecut.tree_structured.sample_generator import SampleGenerator

     gen_dataset = SampleGenerator([3,2,2], allowance_rand=True)
```

To construct the dataset for the randomly generated model, simply invoke the `get_complete_data_set` function found within the `SampleGenerator` class.

```
[2]: x_gen_dataset, y_gen_dataset = gen_dataset.get_complete_data_set()
```

If you also call the `get_complete_data_set` function with an input, as a path to store the result, a file with the input name will be created in the path provided.

```
[3]: x_gen_dataset, y_gen_dataset = gen_dataset.
      ↪get_complete_data_set(file_name="7D_synthetic_data_random")
     print('Generated binary labels:', '\n', y_gen_dataset.astype(int) )
```

```
Generated binary labels:
 [1 1 1 1 1 1 1 1 1 1 1 1 1 1 1 1 1 1 1 1 1 1 1 1 0 0 0 0 0 0 0 0 1 0 1 0 0
 0 0 1 1 0 1 0 0 0 0 1 1 0 1 0 0 0 0 1 1 0 1 0 0 0 0 1 1 0 1 0 0 0 0 1 1 0
 1 0 0 0 0 1 1 0 1 0 0 0 0 1 1 0 1 0 0 0 0 1 1 1 1 1 1 1 1 1 1 1 1 1 1 1 1
 1 1 1 1 1 1 1 1 1 0 0 0 0 0 0 0 0]
```

The randomly set binary function of first-layer black boxes can be taken by calling `get_binary_function_of_box` function of the `SampleGenerator` class. You have to give the ID of first-layer box as an input which is a number in the range `[0, n_box-1]`. Moreover, the randomly set binary function of the output box can be taken by calling `get_binary_function_black_box` of the `SampleGenerator` class. It does not need any input as there is only one output box in the network.

```
[4]: func_0 = gen_dataset.get_binary_function_of_box(0)
     func_1 = gen_dataset.get_binary_function_of_box(1)
     func_2 = gen_dataset.get_binary_function_of_box(2)
     func_bb = gen_dataset.get_binary_function_black_box()
     print('The function of the output-box:', '\n', func_bb )
```

```
The function of the output-box:
 [ True  True False False  True False  True False]
```

You can also obtain the functions of all the first-layer black boxes, along with the function of the output box, simultaneously, by invoking `gen_dataset.print_binary_function_model()`.

```
[5]: gen_dataset.print_binary_function_model()
```

```
Function Box1
([feature_1, feature_2, feature_3]: Binary Output) ->
([0 0 0]: 0), ([1 0 0]: 1), ([0 1 0]: 0), ([1 1 0]: 1), ([0 0 1]: 1), ([1 0 1]:
1), ([0 1 1]: 1), ([1 1 1]: 0)
Function Box2
([feature_4, feature_5]: Binary Output) ->
([0 0]: 0), ([1 0]: 0), ([0 1]: 0), ([1 1]: 1)
Function Box3
```

```
([feature_6, feature_7]: Binary Output) ->
([0 0]: 0), ([1 0]: 1), ([0 1]: 1), ([1 1]: 0)
Function Black Box
([Output_box_1, Output_box_2, Output_box_3]: Binary Output) ->
([0 0 0]: 1), ([1 0 0]: 1), ([0 1 0]: 0), ([1 1 0]: 0), ([0 0 1]: 1), ([1 0 1]:
0), ([0 1 1]: 1), ([1 1 1]: 0)
```

2.2 Generating tree-structured data by setting functions manually

In the same manner as random generating tree-structured data through randomly assigned functions, after importing the `SampleGenerator` class with `allowance_rand=False`, you need to instantiate an object of the class.

[6]:
```
from noisecut.tree_structured.sample_generator import SampleGenerator

gen_dataset = SampleGenerator([3,2,2], allowance_rand=False)
```

To set the functions manually, you can use the `set_binary_function_of_box` function of the `SampleGenerator` class. Input variables of the function are ID of the associated first-layer box and the desired binary function of the box. In the example below, we generated the binary functions depicted in Figure ??.

[7]:
```
gen_dataset.set_binary_function_of_box(0, [0,0,1,1,1,0,1,0])
gen_dataset.set_binary_function_of_box(1, [1,0,1,1])
gen_dataset.set_binary_function_of_box(2, [1,1,0,0])
gen_dataset.set_binary_function_black_box([0,0,1,1,1,0,1,0])
```

After setting all functions of the black boxes, you can check whether your generated data set doesn't provide an in vain black box in the network by calling `has_synthetic_example_functionality` function of the `SampleGenerator` class. If the function returns `Flase`, you might need to change the assigned functions of the black boxes and check it again. This test will enable you to create a non-reducible tree-structured data set by incorporating productive black boxes within the network.

[8]:
```
gen_dataset.has_synthetic_example_functionality()
```

[8]:
```
True
```

You can also get and store the complete data set in the same manner as it has been explained in the previous part.

[9]:
```
x_gen_dataset, y_gen_dataset = gen_dataset.get_complete_data_set()
x_gen_dataset, y_gen_dataset = gen_dataset.
 ↪get_complete_data_set(file_name="7D_synthetic_data_manual")
```

3 Usage Example of NoiseCut

In this section, we present a usage example of `NoiseCut` within the context of a binary classification task. The presented guidelines here can be practically implemented using the `Usage example`

of NoiseCut.ipynb notebook in the package documentation accessible on https://github.com/JRC-COMBINE/NoiseCut/blob/main/docs/notebooks/Usage_example_of_NoiseCut.ipynb.

[1]:
```
import pandas as pd

from noisecut.model.noisecut_model import NoiseCut
from noisecut.tree_structured.data_manipulator import DataManipulator
from noisecut.model.noisecut_coder import Metric
```

3.1 Set training and test sets

Assign X as the features and Y as the labels.

[2]:
```
input_file = '7D_synthetic_data_manual'

data = pd.read_csv(input_file, delimiter='    ', header=None , skiprows=1,␣
↪engine='python')
X = data.iloc[:, :-1]
Y = data.iloc[:, -1]
```

To randomly sample the training and test sets, you can use the build-in function of the DataManipulator class. If you also work with a synthetic dataset (like this example), you can also add noise to the labeling of the data by using get_noisy_data function of the DataManipulator class.

[3]:
```
Training_set_size = 50 # The percentage of training set
Noise_intencity = 5 # The labels' percentage should be toggled from 0 to 1, or␣
↪vice versa.

manipulator = DataManipulator()
x_noisy, y_noisy = manipulator.get_noisy_data(X, Y, percentage_noise =␣
↪Noise_intencity)
x_train, y_train, x_test, y_test = manipulator.split_data(x_noisy, y_noisy,␣
↪percentage_training_data = Training_set_size)
```

```
n_training data is: 64
n_test data is: 64
```

3.2 Fitting the model

To fit the training set into the hybrid model, utilize the NoiseCut class. To instantiate an object of this class, you'll need to provide an input array called n_input_each_box. This array serves as an indicator for the tree structure of the hybrid model. The initial element of n_input_each_box corresponds to the number of input features for the first box in the first layer of the network, which is 3 in the example of the synthetic data generated in the Generation of synthetic data.ipynb notebook; Subsequently, the second element signifies the number of input features for the second first-layer box, which in this case is 2. This pattern continues for the successive elements.

Then, the model can be simply fitted by using fit function of the NoiseCut class.

[4]:
```python
mdl = NoiseCut(n_input_each_box=[3,2,2])
mdl.fit(x_train, y_train)
```

3.3 Evaluation

The evaluation of the NoiseCut algorithm's performance can be conducted by utilizing the test set. This test set can be provided as input to the `predict` function within the `NoiseCut` class.

To assess the model's performance, you can utilize the built-in function of the `Metric` class called `set_confusion_matrix`. This function enables you to establish the confusion matrix, thereby facilitating the computation of accuracy, recall, precision, and F1 score for the predicted output derived from the test dataset.

[5]:
```python
y_predicted = mdl.predict(x_test)

accuracy, recall, precision, F1 = Metric.set_confusion_matrix(y_test,␣
 ↪y_predicted)

print("accuracy = {a:3.3f}, recall = {r:3.3f}, precision = {p:3.3f}, F1 = {f:3.
 ↪3f}".format( a=accuracy, r=recall, p=precision, f=F1 ))
```

```
accuracy = 0.938, recall = 0.974, precision = 0.925, F1 = 0.949
```

3.4 Predictions

The outcomes of the hybrid model can be obtained by calculating the probability of the label being `1` for any binary input fed into the model. This can be accomplished using the `predict_probability_of_being_1` function within the `NoiseCut` class. You can insert a single binary input or even more than one as an array of shape (n_sample, n_festures). If you insert more than one binary input, you receive an array of shape (n_samples,) of the probabilities in one-to-one mapping of the binary input.

[6]:
```python
y_pred_proba = mdl.predict_probability_of_being_1([0,0,0,0,0,0,0])
print(f"Prediction probability for a binary input: {y_pred_proba}")

y_pred_proba = mdl.
 ↪predict_probability_of_being_1([[0,0,0,0,0,0,0],[1,0,1,0,1,0,1]] )
print(f"Prediction probability for two binary inputs: {y_pred_proba}")
```

```
Prediction probability for a binary input: 1.0
Prediction probability for two binary inputs: [1.         0.93333333]
```

The `predict_probability_of_being_1` function can be applied to the complete test set in order to obtain the predicted probabilities. With these probabilities at hand, it becomes possible to calculate the area under the ROC curve.

[7]:
```python
from sklearn import metrics

y_pred_proba = mdl.predict_probability_of_being_1(x_test)
fpr, tpr, thresholds = metrics.roc_curve(y_test.astype(int),  y_pred_proba)
```

```
print('AUC-ROC=', metrics.auc(fpr, tpr) )
```

```
AUC-ROC= 0.9276315789473685
```

3.5 Retrieved functions of the boxes

After fitting model, the predicted binary function of first-layer boxes can be taken by calling `get_binary_function_of_box` of the `NoiseCut` class. You have to give the ID of the first-layer box as an input which is a number in the range `[0, n_box-1]`. Moreover, the predicted binary function of the second-layer box can be taken by calling `get_binary_function_black_box` of the `NoiseCut` class. It does not need any input as there is only one second-layer box.

[8]:
```
func_0 = mdl.get_binary_function_of_box(0)
func_1 = mdl.get_binary_function_of_box(1)
func_2 = mdl.get_binary_function_of_box(2)
func_bb = mdl.get_binary_function_black_box()
func_0
```

[8]:
```
array([False, False,  True,  True,  True, False,  True, False])
```

References

[1] M. Von Stosch, R. Oliveira, J. Peres, and S. F. de Azevedo, "Hybrid semi-parametric modeling in process systems engineering: Past, present and future," *Computers & Chemical Engineering*, vol. 60, pp. 86–101, 2014.

[2] A. P. Teixeira, N. Carinhas, J. M. Dias, P. Cruz, P. M. Alves, M. J. Carrondo, and R. Oliveira, "Hybrid semi-parametric mathematical systems: Bridging the gap between systems biology and process engineering," *Journal of biotechnology*, vol. 132, no. 4, pp. 418–425, 2007.

[3] H. J. Tulleken, "Grey-box modelling and identification using physical knowledge and bayesian techniques," *Automatica*, vol. 29, no. 2, pp. 285–308, 1993.

[4] I. Cascos, "The expected convex hull trimmed regions of a sample," *Computational Statistics*, vol. 22, no. 4, pp. 557–569, 2007.

[5] B. Fiedler and A. Schuppert, "Local identification of scalar hybrid models with tree structure," *IMA Journal of Applied Mathematics*, vol. 73, no. 3, pp. 449–476, 2008.

[6] A. A. Schuppert, "Extrapolability of structured hybrid models: a key to optimization of complex processes," in *Equadiff 99: (In 2 Volumes)*, pp. 1135–1151, World Scientific, 2000.

[7] M. E. Samadi, S. Kiefer, S. J. Fritsch, J. Bickenbach, and A. Schuppert, "A training strategy for hybrid models to break the curse of dimensionality," *Plos one*, vol. 17, no. 9, p. e0274569, 2022.

[8] K. L. Pudenz and D. A. Lidar, "Quantum adiabatic machine learning," *Quantum information processing*, vol. 12, no. 5, pp. 2027–2070, 2013.

[9] H. Neven, V. S. Denchev, G. Rose, and W. G. Macready, "Training a binary classifier with the quantum adiabatic algorithm," *arXiv preprint arXiv:0811.0416*, 2008.

[10] A. Billionnet, “Solving a cut problem in bipartite graphs by linear programming: Application to a forest management problem,” *Applied mathematical modelling*, vol. 34, no. 4, pp. 1042–1050, 2010.

[11] T. F. Edgar, D. M. Himmelblau, and L. S. Lasdon, “Optimization of chemical processes,” *(No Title)*, 2001.
